# Decreased echinocandin susceptibility in *Candida parapsilosis* causing candidemia and emergence of a pan-echinocandin resistant case in China

**DOI:** 10.1080/22221751.2022.2153086

**Published:** 2022-12-24

**Authors:** Yating Ning, Meng Xiao, David S. Perlin, Yanan Zhao, Minya Lu, Yi Li, Zhengyu Luo, Rongchen Dai, Shengjie Li, Jiajun Xu, Lingli Liu, Hong He, Yun Liu, Fushun Li, Yuguang Guo, Zhongju Chen, Yingchun Xu, Tianshu Sun, Li Zhang

**Affiliations:** a Department of Laboratory Medicine, State Key Laboratory of Complex Severe and Rare Diseases, Peking Union Medical College Hospital, Chinese Academy of Medical Sciences and Peking Union Medical College, Beijing, People’s Republic of China; b Graduate School, Chinese Academy of Medical Sciences and Peking Union Medical College, Beijing, People’s Republic of China; c Beijing Key Laboratory for Mechanisms Research and Precision Diagnosis of Invasive Fungal Diseases, Beijing, People’s Republic of China; d Center for Discovery and Innovation, Hackensack Meridian Health, Nutley, NJ, USA; e School of Public Health, Zhejiang Chinese Medical University, Hangzhou, People’s Republic of China; f Medical Research Centre, State Key Laboratory of Complex Severe and Rare Diseases, Peking Union Medical College Hospital, Chinese Academy of Medical Science, Beijing, People’s Republic of China; g Department of Clinical Laboratory, the Affiliated Hospital of Qingdao University, Qingdao, People’s Republic of China; h Department of Laboratory Medicine, Changhai Hospital, Second Military Medical University, Shanghai, People’s Republic of China; i Department of Laboratory Medicine, The First Hospital of China Medical University, Shenyang, People’s Republic of China; j Department of Laboratory Medicine, Liaoning Provincial People’s Hospital, Shenyang, People’s Republic of China; k Department of Laboratory Medicine, Tongji Hospital, Tongji Medical College, Huazhong University of Science and Technology, Wuhan, People’s Republic of China

**Keywords:** *Candida parapsilosis*, echinocandin resistance, breakthrough candidemia, *FKS1*, S656P

## Abstract

*Candida parapsilosis* is becoming a predominant non-*albicans* cause of invasive candidiasis (IC). Echinocandins are the preferred choice for IC treatment and prophylaxis. Resistance to echinocandins in *C. parapsilosis* has emerged in several countries, but little is known about the susceptibility profile in China or about mechanisms of resistance. Here, we investigated the echinocandin susceptibilities of 2523 *C. parapsilosis* isolates collected from China and further explored the resistance mechanism among echinocandin-resistant isolates. Anidulafungin exhibited the highest MICs (MIC_50/90_, 1 and 2 µg/mL; GM, 0.948 µg/mL), while caspofungin showed better activity (0.5 and 1 µg/mL; 0.498 µg/mL). Significantly higher echinocandin MICs were observed among blood-derived isolates compared to others, especially for caspofungin (GM, 1.348 µg/mL vs 0.478 µg/mL). Isolates from ICU and surgical wards also showed higher MICs. Twenty isolates showed intermediate phenotypes for at least one echinocandin. One was resistant to all three echinocandins, fluconazole and voriconazole, which caused breakthrough IC during long-term exposure to micafungin. WGS revealed this isolate carried a mutation S656P in hotspot1 region of Fks1. Bioinformatics analyses suggested that this mutation might lead to an altered protein conformation. CRISPR Cas9-mediated introduction of this mutation into a susceptible reference *C. parapsilosis* strain increased MICs of all echinocandins 64-fold, with similar results found in the subspecies, *C. orthopsilosis* and *C. metapsilosis*. This is the first report of a multi-azole resistant and pan-echinocandin resistant *C. parapsilosis* isolate, and the identification of a *FKS1*^S656P^ conferring pan-echinocandin resistance. Our study underscores the necessity of rigorous management of antifungal use and of monitoring for antifungal susceptibility.

## Introduction

*Candida* species are among the most common causative agents of invasive infections, especially in patients in intensive care. Indeed, approximately 35% to 51% of invasive candidiasis (IC) cases occurred in intensive care units (ICU), resulting in a mortality rate between 40% and 51% [1,2]. *Candida albicans* accounts for the majority of IC worldwide; however the combined incidence of invasive non-*albicans* IC has risen markedly in recent decades [3–5]. Among non-*albicans Candida* spp., *C. parapsilosis* is of particular concern clinically, and depending on geography, patient age and other factors, it may rank as the first or second-leading cause of IC [4,6,7]. Patients under total parenteral nutrition or with invasive medically implanted devices and low-birth-weight neonates are among those at the highest risk of infections by this species [8]. Despite the prevalence of this pathogenic fungus, treatment options are restricted to only a few antifungal drug classes [9]. In the last five years, this predicament has been exacerbated by the increasing surge of *C. parapsilosis* resistant to azoles, which have been widely used for the treatment of IC and for prophylaxis in many countries [10–14].

Another major antifungal class is echinocandins, including caspofungin, micafungin, and anidulafungin. These compounds noncompetitively inhibit β-1,3-D-glucan synthase, thus interfering with the biosynthesis of β-1,3-D-glucan, a key fungal-specific component of the cell wall [15]. Since echinocandins have low renal or hepatic toxicity and few serious drug–drug interactions, they are recommended as a first-line therapy for IC and as prophylaxis for patients undergoing hematopoietic stem cell transplantation [16]. Importantly, multiple studies have demonstrated that echinocandins represent an efficacious remedy for the treatment of azole-resistant *C. parapsilosis* isolates [17,18].

Resistance to echinocandins has been rarely observed among most *Candida* species, although *C. glabrata* shows a greater propensity to develop resistance [15]. However, the alarming emergence of echinocandin-resistant *C. parapsilosis* clinical isolates has been reported in Iran, Turkey, America, and Spain [19–22]. Notably, little is known about echinocandin susceptibility in China. Echinocandin resistance in *Candida* spp. is mainly due to the occurrence of mutations in the hot-spot (HS) regions of *FKS* genes, which encode the catalytic subunits of β-1,3-D-glucan synthase. Uniquely among *Candida* spp., *C. parapsilosis* manifests intrinsically higher *in vitro* minimal inhibitory concentrations (MIC) to echinocandins, owing to a natural amino acid polymorphism P660A occurring in Fks1 [23]. To date, only one mutation F652S in HS1 of Fks1 has been identified specifically in an pan-echinocandin *C. parapsilosis* clinical isolate but not in any susceptible isolates, while R658G specifically in micafungin mono-resistant isolates, and one heterozygous mutation, F1386S outside of HS, in an anidulafungin mono-resistant clinical isolate [19,22,24]. However, no direct experimental evidence has been reported to confirm the function of these mutations, and there remains little known about the resistance mechanisms of *C. parapsilosis*.

Herein, we investigated the *in vitro* echinocandin susceptibility of 2523 invasive *C. parapsilosis* isolates collected by CHIF-NET 2015–2019 programme from 87 hospitals in China. We identified one clinical isolate causing breakthrough IC during long-term exposure to micafungin. This isolate was determined to be resistant to all echinocandins, fluconazole and voriconazole. Through whole genome sequencing (WGS) analyses and analyses of mutations generated by site-directed CRISPR Cas9 technology, we further explored the mechanism of echinocandin resistance in *C. parapsilosis* complex. To the best of our knowledge, this is the first report of a pan-echinocandin resistant *C. parapsilosis* clinical isolate harbouring S656P in *FKS1* that was the cause of breakthrough IC, and the confirmation of the resistance function of Fks1^S656P^.

## Materials and methods

### Isolates and identification

Clinical isolates were made available by the China Hospital Invasive Fungal Surveillance Net (CHIF-NET 2015–2019, August 2014 to July 2019) project, and identified to subspecies level by DNA sequencing of the internal transcribed spacer region [25]. Echinocandin-susceptible type strains *C. parapsilosis* sensu stricto ATCC 22019, *C. orthopsilosis* ATCC 96139 and *C. metapsilosis* ATCC 96143 were used as parental strains in CRISPR experiments for functional verification of the specific *FKS1* mutations. All clinical isolates and strains were stored in 40% glycerol at −80°C, and the fungi were routinely cultured in YPD media (1% yeast extract, 2% dextrose and 2% peptone) at 30°C unless otherwise indicated. The program was approved by the Human Research Ethics Committee of Peking Union Medical College Hospital (S-263).

### Drug susceptibility determination

*In vitro* susceptibilities to three echinocandins (micafungin, caspofungin and anidulafungin) were determined for 2523 isolates, and the susceptibilities of TJ1197 to azoles, 5-flucytosine and amphotericin B, were determined using the broth microdilution method according to the Clinical and Laboratory Standards Institute (CLSI) M27 document [26]. MIC values were interpreted according to CLSI M27M44S and M57S, supplemented by the reported of Canton's study [27–29]. Isolates with MIC values of 4 µg/mL were defined as intermediate to echinocandins, while ≥8 µg/mL were considered to indicate resistance to both echinocandins and fluconazole. MICs ≥1 µg/mL were considered as resistance in case of voriconazole.

### Detection of other phenotypes

Biofilm formation was analyzed as previously described, and other phenotypical analyses were performed essentially as described but with minor modifications [30]. Colony morphologies were observed after 3 days of incubation on SDA agar incubated at 37°C, and then colonies were washed off to analyze the development of agar invasion. A single colony was incubated in YPD broth overnight at 37°C with agitation at 200 rpm, and aggregation and cell morphologies were then observed.

### WGS and single nucleotide polymorphism (SNP) analyses

A single colony of the selected isolate was grown in YPD liquid medium for overnight at 30°C, 200 rpm. Genomic DNA extraction was performed using the QIAamp DNA Mini Kit (Qiagen, Hilden, Germany). A paired-end DNA library was constructed with the NEBNext® Ultra™ DNA Library Prep Kit (NEB, Massachusetts, USA), then sequenced using the Illumina NovaSeq platform with in the PE150 (2×150 bp reads) mode. Raw FASTQ files were processed through a standard pipeline consisting of low-quality read filtering through Trimmomatic (version 0.36). Reads were mapped against the antifungal drug-susceptible *C. parapsilosis* ATCC 22019 reference genome using bowtie2 (v2.3.5.1). SNPs were analyzed using SAMtools (v1.3.1) and VarScan (v2.4.3) [31,32]. The filter setting for SNPs included minimum read depth at a position (>10), minimum supporting reads (>2), and minimum frequency to call homozygote (>0.75). SNP annotations were performed with SnpEff (v4.2) as previously described [33]. Reads have been deposited in the NCBI Sequence Read Archive under BioProject accession number PRJNA905552.

### Bioinformatics prediction

Transmembrane helix predictions were performed with the DeepTHMMM bioinformatic tool [34]. The biological effects of the protein mutation were predicted by the PROVEAN tool, and protein stability changes were predicted with the VAPOR tool [35]. The three-dimensional (3D) models for protein structures of the Fks1 variants were obtained by modelling using the I-TASSER online server [36]. Structures were visualized in PyMOL 2.5.0.

### Construction of site-directed mutants

*C. parapsilosis* complex site-directed mutagenesis was performed as previously reported with a minor modification [37]. Repair templates were obtained by primer extension of overlapping oligonucleotides (Table S1), which were designed for both the mutation of the target base and synonymous mutation of the PAM site to protect the donor sequence from being clipped by Cas9. Competent yeast cells and transformations were performed with the Frozen-EZ Yeast Transformation II Kit (Zymo Research, California, USA). Purified repair template (2.5 μg) and a pCP-tRNA plasmid (2.5 μg) carrying *CAS9* and the sgRNA coding sequence were mixed with approximately 50 μL of cells prior to transformation. Then, recovered cells were plated on YPD plates containing 200 µg/mL nourseothricin. Mutants were confirmed through Sanger sequencing of the nourseothricin-resistant transformants.

### RNA extraction and gene expression analysis

Single colonies were cultured in YPD broth at 37°C overnight. The OD_600_ of cultures was adjusted to 0.2 in fresh YPD broth lacking micafungin or supplemented with the sub-MIC concentration of micafungin, 32 µg/mL for TJ1197 and ATCC 22019_*FKS1*^S656P^, and 0.5 µg/mL for TJ1197_*FKS1*^WT^ and ATCC 22019. These samples were incubated at 37°C with agitation at 200 rpm for 4 h. RNA was extracted as previously described [38]. Quantitative PCR was performed using TB Green® Premix DimerEraser™ (Takara, Shiga, Japan) to measure expression levels of the *FKS1* gene, with the *ACT1* gene serving as an internal control [20]. Primers are shown in Table S1. Experiments were carried out in three biological and two technical replicates.

### Statistical analysis

All statistical analyses were performed with GraphPad Prism 8 software. Nonparametric Mann–Whitney tests were performed to compare the MIC (log2(MIC values)) differences of between isolates of different samples or wards. Statistical analyses of gene expression, and absorbance values were carried out using the unpaired Student's t test. The differences were considered statistically significant with a *P* value < 0.05.

## Results

### Distribution of susceptibility among clinical isolates

**Figure 1.**
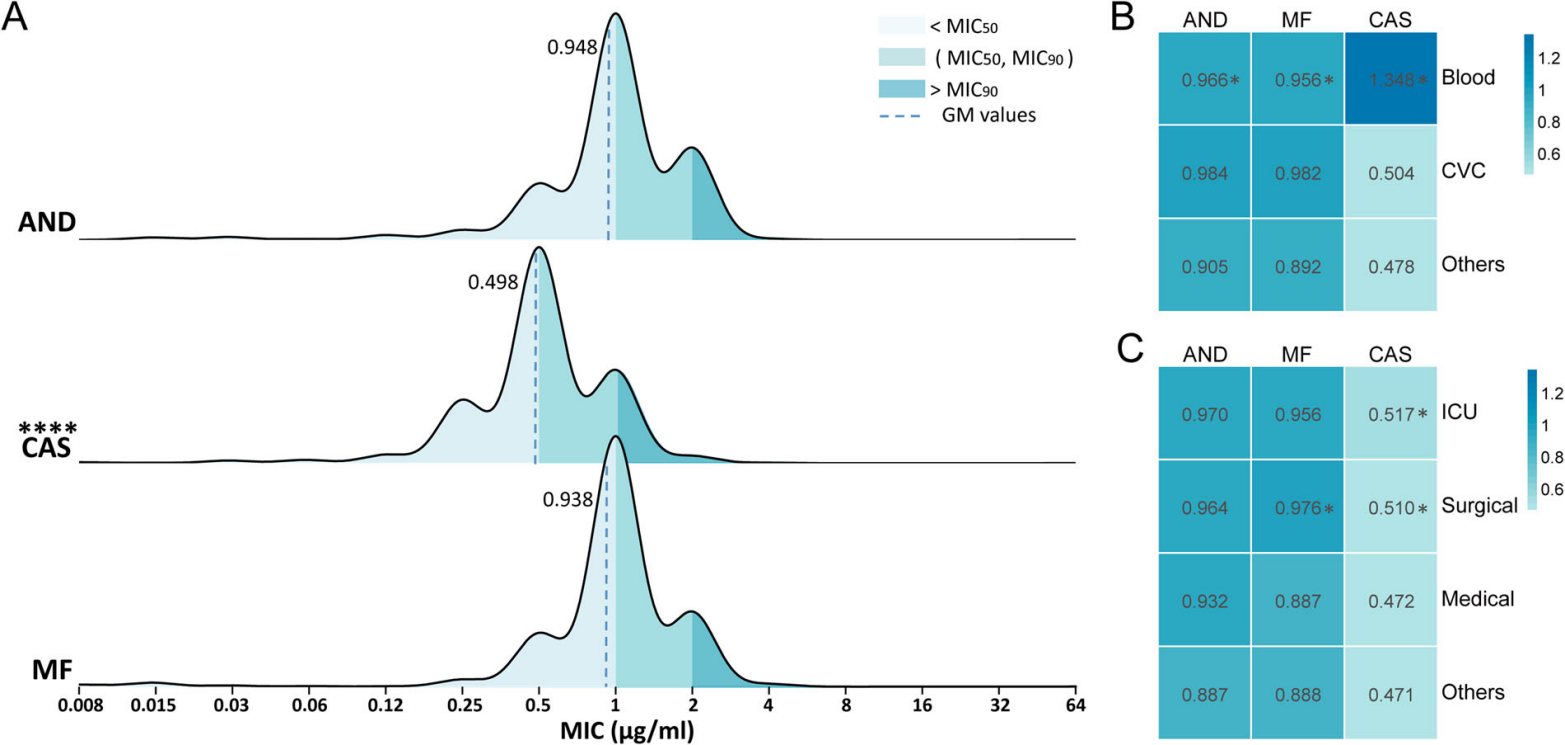
Profile of *C. parapsilosis* susceptibility to echinocandins. (A) Distribution of susceptibility to three echinocandins of 2523 clinical isolates from China collected by CHIF-NET 2015–2019 programme. (B and C) Susceptibility differences of isolates regarding different samples (B), and hospital departments (C). Geometric mean of the MIC (GM) values are shown in the boxes; differences between each two groups were analyzed, while only significant differences compared to the “others” group are shown in the figure. AND, anidulafungin; CAS, caspofungin; MF, micafungin. *, *P* < 0.05; ****, *P* < 0.0001.

Between August 2014 and July 2019, 2523 *C. parapsilosis* clinical isolates from 87 hospitals, 29 provinces in China, were submitted to the CHIF-NET programme as part of invasive fungal disease surveillance. Of three echinocandins tested, anidulafungin exhibited the highest MIC values (MIC range, 0.015–64 µg/mL; geometric mean MIC (GM), 0.948 µg/mL), followed by micafungin (MIC range, 0.008–64 µg/mL; GM, 0.938 µg/mL). Both of these two drugs displayed the same MIC_50_ of 1 µg/mL and MIC_90_ of 2 µg/mL. The *in vitro* activity of caspofungin was significantly better than those of the other two echinocandins (*P* < 0.0001), with MIC_50/90_ values of 0.5/1 µg/mL, a GM of 0.498 µg/mL and MIC ranging from 0.008 to 64 µg/mL (Figure 1A).

Of various specimen types, 57.8% (*n* = 1458) of the *C. parapsilosis* isolates were recovered from blood. Significantly higher MICs of echinocandins were observed among isolates separated from blood specimens compared to those from other specimens (*n* = 796, 31.5%) except for central venous catheters (CVC) (*n* = 269, 10.7%). Of note, when comparing the GM values for blood-derived isolates with the other-derived, caspofungin exhibited the largest difference (1.348 µg/mL vs 0.478 µg/mL, *P *< 0.05), while those of anidulafungin were 0.966 and 0.905 µg/mL (*P *< 0.05), and micafungin were 0.956 and 0.892 µg/mL (*P *< 0.05). A dilution gradient difference of the caspofungin GM of over 1 also occurred between blood-derived and CVC-derived isolates, but the difference was not considered statistically significant (1.348 µg/mL vs 0.504 µg/mL, *P *= 0.8295) (Figure 1B).

In terms of susceptibility distribution by department, isolates recovered from patients in the ICU (*n* = 691, 27.4%) were of equal susceptibility to echinocandins as those from patients in the surgical ward (*n* = 944, 37.4%). Both ICU- and surgical-derived isolates showed higher overall MICs compared to other department-derived isolates; this effect was apparent with caspofungin (GM, 0.517 µg/mL (ICU) / 0.510 µg/mL (surgical) vs 0.472 µg/mL, *P *< 0.05) (Figure 1C).

Among the 2523 isolates, 99.64%, 99.25%, and 99.80% were susceptible to anidulafungin, micafungin, and caspofungin, respectively. Twenty (0.79%) isolates showed an intermediate phenotype for at least one echinocandin, of which six isolates were intermediate to micafungin and anidulafungin, two were intermediate to micafungin and caspofungin, and one showed intermediate susceptibility to all echinocandins. Notably, one isolate, TJ1197, was determined to be resistant to all three echinocandins.

### Case characteristics of a breakthrough candidemia caused by the pan-echinocandin resistant C. parapsilosis

The patient, a 7-week male infant, had intermittent fever without obvious incentive that reached a peak temperature of 39.5°C. He then received antibiotic treatment with potassium amoxicillin clavulanate and cefazolin in another hospital. Three days after the antibiotic therapy, he suffered severe diarrhea, with more than ten episodes per day, coupled with vomiting. Two days later, he was transferred to the pediatric ICU of the present hospital, but no pathogen was identified. In the ICU, a peripherally inserted central catheter was placed and used to deliver empirical antimicrobial treatments including micafungin, vancomycin and levofloxacin. Cortisone therapy and supportive treatments were also administered.

Six days after admission, the patient developed massive bloody stools, and subsequently underwent ileostomy. In the meantime, total parenteral nutrition was administered. One month after ileostomy, he was of extremely low weight and malnourished. Despite continued antimicrobial therapy with micafungin and broad-spectrum antibiotics, his fever persisted. Approximately 50 days after admission and more than one month after administration of micafungin, blood cultures indicated the presence of fungal spores. Antibiotic treatments were then changed to voriconazole.

Five days later, *C. parapsilosis* (TJ1197) grew from blood cultures, and *in vitro* susceptibility tests revealed that this isolate carried a resistance phenotype for both fluconazole and voriconazole, and of wild-type phenotype for amphotericin B, 5-flucytosine and itraconazole. Given that the patient could not tolerate amphotericin B or itraconazole due to liver dysfunction, voriconazole treatment was maintained additionally combined with 5-flucytosine. Ten days later the patient remained in poor condition and was discharged voluntarily (Table 1 & Figure S1).

**Table 1.** Clinical characteristics of a patient with breakthrough candidemia caused by pan-echinocandin resistant *C. parapsilosis.*

Characteristics	Patient infected with TJ1197
Age at admission	7 weeks
Gender	Male
Department of admission	Pediatric ICU
Reason for admission	Intermittent fever
Admitting diagnosis	Pulmonary infection and severe diarrhea
Site of isolation	Blood
Clinical status at time of positive culture	
Prematurity	NO
Immunosuppressive state	YES
Neutropenia (<10^9^/L)	NO
Catheter insertion	YES
Indwelling urinary catheter	YES
Total parenteral nutrition	YES
Abdominal surgery within 30 days	YES; ileostomy
Broad-spectrum antibiotics	YES
Prior antifungal treatment	YES; micafungin, 45 days
Prolonged ICU stay	YES; 45 days
Therapy & Outcome	
CVC removal	NO
Antifungal therapy after culture	Voriconazole and 5-flucytosine
Outcome	Poor condition and discharged voluntarily
Characteristics of the clinical isolate TJ1197*	
Antifungal resistance	Echinocandins, fluconazole, voriconazole
Colony morphology	Crepe
Cell shape	Pseudohyphal and yeast-form cells
Aggregation in liquid culture	High^#^
Biofilm formation	Strong^#^
Agar invasiveness	High^#^

*See Figure S1 for detail; ^#^Compared to ATCC 22019.

### Potential resistance mechanisms revealed by WGS analysis

**Figure 2.**
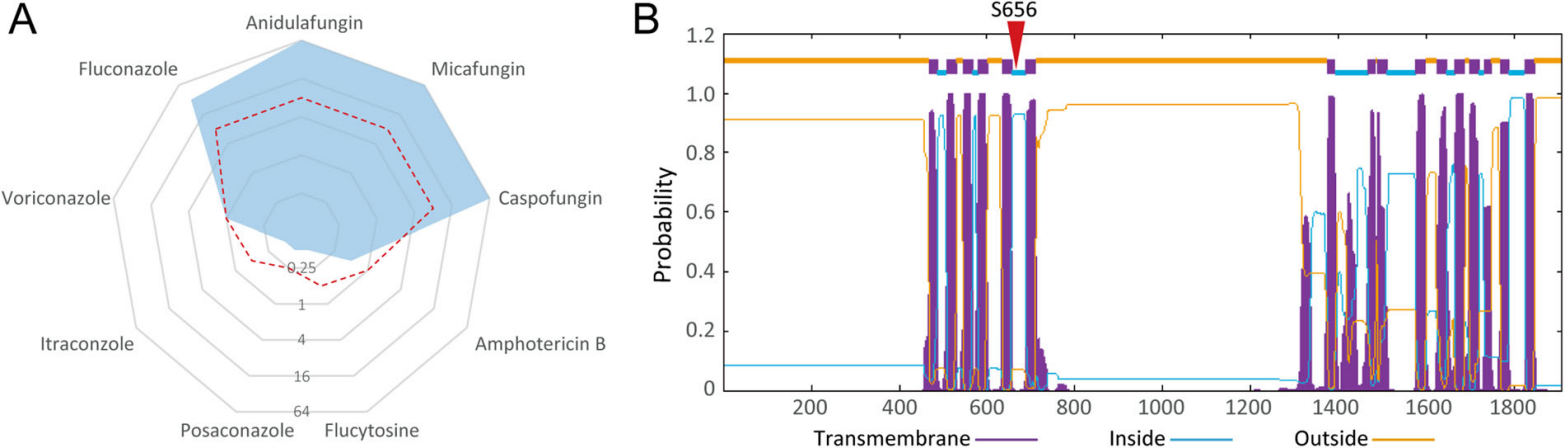
Strain characteristics of the pan-echinocandin resistant *C. parapsilosis* clinical isolate. (A) *In vitro* susceptibility to nine common antifungal drugs. The dashed red line indicates the breakpoints for defining drug resistance or cut-off values for non-wild type. (B) Transmembrane helix predictions for Fks1 of *C. parapsilosis.* The location of the amino acid 656 is labelled.

Our group repeated susceptibility tests for clinical isolate TJ1197 using the broth microdilution method according to CLSI M27 [26]. Our results verified that this isolate displayed resistance to all three echinocandins with uniform MIC values of 64 µg/mL, and also cross-resistance to fluconazole (32 µg/mL) and voriconazole (1 µg/mL), which have similar short chain structures (Figure 2A).

Since resistance to antifungal drugs is often attributed to single-nucleotide variations of genes encoding drug targets and relevant regulatory pathways [15], we performed WGS and SNP analyses of TJ1197 through mapping reads against the reference genome of *C. parapsilosis* ATCC 22019 strain, which is susceptible to both azoles and echinocandins. Across genes reported in echinocandin-resistance in *Candida* pathogens, we identified one point missense mutation, T1966C, harboured in *FKS1*, which encodes the catalytic subunit of β-1,3-D-glucan synthase. This mutation results in the change of amino acid serine at position 656 to proline (S656P). A protein topology prediction analysis indicated amino acid 656 is located in the HS1 region within the intracellular half of the fifth transmembrane helix of Fks1 (Figure 2B).

We also identified two missense mutations associated with azole resistance. One of these mutations was G1193T (R398I) in *ERG11*, which encodes the azole target enzyme sterol 14-demethylase. The second was T1654C (Y552H) in *MRR1,* a transcriptional factor of multidrug efflux pumps, including *MDR1*.

### The S656P mutation in Fks1 confers pan-echinocandin resistance in C. parapsilosis

**Figure 3.**
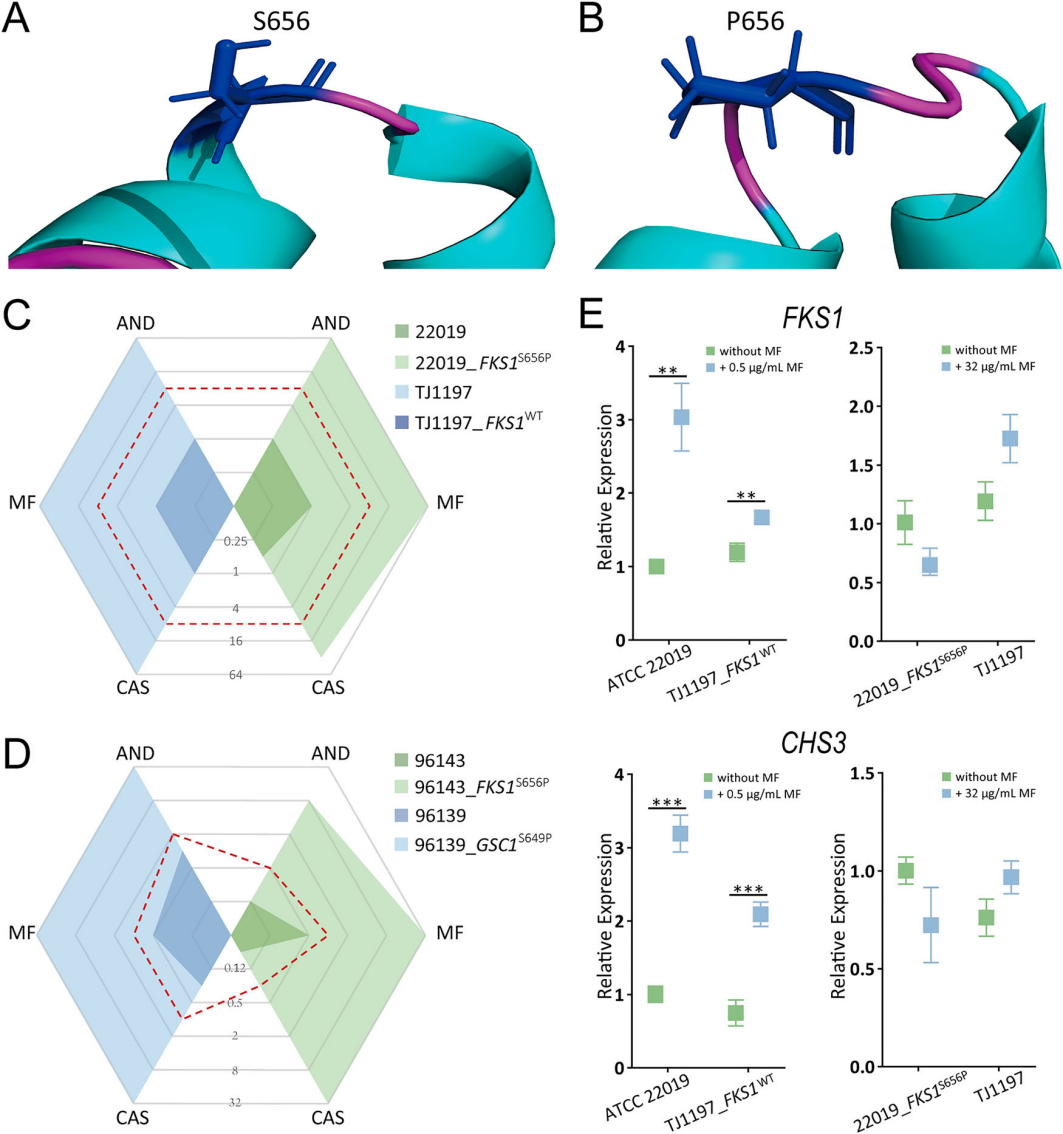
Structural and functional effect of the S656P substitution in Fks1. (A) The predicted structural model for the variants of the Fks1 protein (amino acids 400∼900), the S656P substitution would result in the helix-to-coil transition, disrupting conformation of this ɑ-helix. (B) Echinocandin MICs for susceptible *C. parapsilosis* strain ATCC 22019 and its mutant harbouring Fks1 S656P, and the pan-echinocandin resistant clinical isolate TJ1197 and its mutant with WT of Fks1. (C) Changes in expression of the *FKS1* and *CHS3* genes in response to micafungin at the sub-MICs. (D) Echinocandin MICs for susceptible *C. orthopsilosis* ATCC 96139, *C. metapsilosis* ATCC 96143, and their mutants carrying homologous modifications, S649P and S656P, respectively. Dashed red lines indicate the breakpoints for defining drug resistance or cut-off values for non-wild type. AND, anidulafungin; CAS, caspofungin; MF, micafungin. **, *P* < 0.01; ***, *P* < 0.001.

In order to investigate the implications of the S656P replacement in Fks1, we used the PROVEAN tool to predict its effect on protein's biological function. This analysis characterized the S656P alteration as a deleterious mutation, with a score of −4.827. And the free energy of Fks1^S656P^, predicted by the VAPOR tool, was reduced relative to that of the wild type protein (ΔΔG: −1.27, based on MUpro; −0.43, based on I-Mutant 2.0). This decrease suggests that such a substitution reduces the stability of the protein structure. In addition, the predicted 3D structure showed the S656P alteration would result in a helix-to-coil transition and a potential conformational change (Figure 3A&B).

We next explored the potential direct impact of this mutation on echinocandin resistance. To perform that, the wild-type base (T) was introduced into the echinocandin pan-resistant clinical isolate TJ1197 to create the homozygous mutant strain TJ1197_*FKS1*^WT^ utilizing a plasmid based CRISPR-Cas9 system [37]. Interestingly, MICs of TJ1197_*FKS1*^WT^ for all echinocandins were decreased 64-fold, and the echinocandin susceptibility profile changed from resistant to susceptible. Conversely, after introducing the mutant-type base (C) into the susceptible background ATCC 22019, 64-fold elevations were observed in MICs of all three echinocandins (Figure 3C)*.* Given that Fks1 S656 is highly conserved among members of the *C. parapsilosis* complex, we further investigated the universality of the pan-echinocandin resistance effect of the S656P mutation in two other subspecies, *C. orthopsilosis* and *C. metapsilosis.* Introduction equivalent mutation S649P of Glycan synthase complex 1 (Gsc1), the *C. orthopsilosis* ortholog of Fks1, into the susceptible *C. orthopsilosis* strain ATCC 96139 was observed to increase MIC 32-, 64-, 128-fold to anidulafungin, micafungin, and caspofungin, respectively. Analogously, after introducing the equivalent mutation, S656P, into *C. metapsilosis* ATCC 96143, 64-fold, 64-fold and 512-fold increases in MIC to anidulafungin, micafungin and caspofungin, respectively, were observed (Figure 3D).

Previous studies have shown that *Candida* species can rapidly respond to echinocandin treatment with a compensatory elevation in chitin content; this response protects the yeast cells from cell wall damage that would occur due to the inhibition of β-1,3-D-glucan synthesis [39,40]. To explore changes to this response caused by the S656P mutation in Fks1, we used qRT-PCR to measure the expression levels of *FKS1* and the gene encoding chitin synthase (*CHS3*) with or without sub-MIC of micafungin treatment. Micafungin treatment of these strains ATCC 22019 and TJ1197_*FKS1*^WT^, which have wild type of *FKS1*, led to 3.2- and 1.4-fold increases in *FKS1* expression, as well as 3.2- and 2.8-fold increase in *CHS3* expression, respectively (all *P *< 0.01 relative to untreated). In contrast,the mutant strain ATCC 22019_*FKS1*^S656P^ and the clinical isolate TJ1197, both of which carry the S656P mutation, showed no significant changes in the expression levels of either *FKS1* or *CHS3* (Figure 3E). These results support that S656P in *FKS1* is sufficient to protect *C. parapsilosis* against echinocandins at sub-MIC.

## Discussion

*C. parapsilosis* has rapidly become a predominant species causing IC behind *C. albicans* [8]. Unfortunately, a surge of azole resistance in *C. parapsilosis* has complicated antifungal treatment options, and echinocandins have become the preferred choice for treatment of IC and antifungal prophylaxis [10–15]. Overall, while identified with some frequency in *C. glabrata*, echinocandin resistance had occurred only rarely to *Candida* species. Yet, recently, the emergence of echinocandin-resistant *C. parapsilosis* has posed an alarming threat [19,20,41–43].

Considering the fact that echinocandins have been approved for clinical treatment in China since 2006, it is imperative to surveil susceptibility profiles of local clinical isolates. In this study, we analyzed a total of 2523 *C. parapsilosis* isolates collected by CHIF-NET obtained from 87 Chinese hospitals. Consistent with other surveillance data, we found that these *C. parapsilosis* isolates from China exhibited considerably high MICs but low resistance (0.03%) to echinocandins (Figure 1A) [41,43]. High MICs have been attributed to the natural mutation P660A occurring in HS1 of Fks1 protein [23]. Of note, caspofungin showed significantly better *in vitro* activity against the *C. parapsilosis* isolates tested in our study than both micafungin and anidulafungin (Figure 1A), consistent with reports from global surveillance programmes [43–46]. This trend was also observed in *C. guilliermondii*, another *Candida* specie possessing a phenotype of inherently high MICs to echinocandins [47]*.* Other common *Candida* species, including *C. albicans, C. tropicalis, C. glabrata* and *C. lusitaniae,* generally were most susceptible to micafungin among the three echinocandins, while *C. krusei and C. pelliculasa* most susceptible to anidulafungin [41,43,44,47]. Therefore, three echinocandins exhibited species-specific susceptibility tendencies. This specificity is likely caused by differences in the structure or composition of the cell wall between species but may also reflect cellular drug tolerance mechanism. Regarding drug structures, both micafungin and anidulafungin have cyclic side chains; specifically, micafungin has a 3,5-diphenyl-substituted isoxazole ring chain, while anidulafungin is characterized by an octyloxytriphenyl chain. Caspofungin, one the other hand, has a unique dimethylmyristoyl side chain that is instrumental for its intercalation into the fungal cell wall; this anchoring chain may lead to the higher susceptibility of *C. parapsilosis* [48]. However, the relationships among chemical structures and antifungal activities, and the reasons for the species-specific phenotypes need to be explored further.

Attention should also be drawn here to the significantly higher MICs of all echinocandins in blood-derived *C. parapsilosis* isolates compared those from other samples (Figure 1B). Multiple studies have demonstrated that serial exposure to echinocandins can lead to decreased susceptibility, and it is also an important risk factor for the resistance development in *Candida* species [42,49,50]. Due to the large molecular weight and low oral bioavailability of echinocandins, they can only be administered intravenously, and this route of administration makes the blood the site of the highest drug concentration among human tissues [51]. Therefore, strains present in the blood were under elevated selection pressures, which may be partially responsible for the higher echinocandin MICs in blood-derived isolates. Notably, caspofungin exhibited the largest difference of GM values between blood- and nonblood-derived *C. parapsilosis* in our study; yet, the reasons for this phenomenon are unclear. Moreover, decreased susceptibility was also found in isolates from patients in ICU wards, which may be due to the high frequency of echinocandin use. These phenomena underscore the necessity of surveilling antifungal use and susceptibility in clinical settings.

Recently, *C. parapsilosis* was described as the most common species responsible for in breakthrough IC among patients receiving echinocandin therapy [52]. In our study, a breakthrough infection occurred due to the pan-echinocandin resistant *C. parapsilosis* during long-term exposure to micafungin. Cornely et al. have attributed the occurrence of breakthrough invasive fungal infections (IFI) to three aspects: individual host characteristics, fungal characteristics, and iatrogenic factors [53]. In our case, the patient was associated with several factors that predicted a high risk of acquiring breakthrough IFI, including immunosuppression, gastrointestinal surgery (ileostomy), exposure to at least 2 antibiotics over 14 days and an extended stay in the ICU. Colonization of gastrointestinal tract or skin by the pathogen is the first step in the pathogenesis of IC. Importantly, a recent study demonstrated that *C. parapsilosis* stably colonized the gut in patients receiving micafungin for antifungal prophylaxis, due to its inherently higher MICs [54]. Thus, the use of echinocandins could promote expansion of *C. parapsilosis* species through reducing commensal microflora, thus increasing the frequency of breakthrough infections [42,54]. Meanwhile, *C. parapsilosis* is known to grow rapidly in total parenteral nutrition, increasing proportion of this organism in the gut microbiome [8,55]. Taken together, all these predisposing factors from our isolated *C. parapsilosis*, including its echinocandin resistance, biofilm formation, and virulence traits facilitating adherence and adaptation to microenvironments, rendered patient susceptible to breakthrough infection.

We identified a *C. parapsilosis* clinical isolate with pan-echinocandin resistance and cross-resistance to azoles. It is well known that mutations in *FKS* genes confer echinocandin resistance in *Candida* species, and these typically occur in two specific and highly conserved regions of the Fks1 protein, called HS1 and HS2 [15]. In *C. parapsilosis*, these regions span amino acids 652 and 660 (HS1), as well as 1369 and 1376 (HS2). The substitution P660A in Fks1 HS1 has been proven to account for the intrinsically higher MICs to echinocandins of *C. parapsilosis*, although it is unable to confer resistance [20,23]. Prior to our study, several mutations of Fks1 had been identified in *C. parapsilosis* clinical isolates, but only three of these mutations specifically occurred in resistant isolates: R658G harboured in a micafungin mono-resistant isolate, F652S harboured in a pan-echinocandin isolate and heterozygous F1386S in an anidulafungin mono-resistant isolate [19,22,24]. However, all of these findings await functional verification.

In this study, a novel pan-echinocandin resistant *C. parapsilosis* clinical isolate carrying a S656P homozygous mutation was identified. This mutation is located in HS1 of Fks1, and predicted to within the intracellular half of the fifth transmembrane helix of this protein. A heterozygous mutation of S656P was previously reported in a *C. parapsilosis* strain which acquired resistance through an *in vitro* anidulafungin-evolution experiment. This strain exhibited echinocandin resistance profiles similar to that of our clinical isolate, in that both strains had MICs of greater than 8 µg/mL to the three echinocandins [50]. Amino acid 656, located in HS1 of *C. parapsilosis*, Fks1, is highly conserved among *Candida* species, and studies have reported that a homologous modification of S645 in *C. albicans* Fks1 leads to the most pronounced resistance phenotype and accounts for major resistance. Similarly, the homologous mutation S629 of Fks2 confers the highest MICs in *C. glabrata* [15,56]. While loss of sterol desaturase activity due to the G111R mutation in Erg3 also results in reduced susceptibility to micafungin and decreased susceptibility to anidulafungin and caspofungin of *C. parapsilosis* [21]*,* we did not identify any mutations in *ERG3* gene in this isolate.

Among all proteinogenic amino acids, proline, due to its unique pyrrolidine ring side chain without the NH group, is the only one not permissive of forming main-chain hydrogen bonds. This structural rigidity becomesa large kinetic barrier between cis and trans conformations of the peptide bond [57]. The “deleterious” result of the S656P change predicted by the PROVEAN tool suggested that this region might have a relevant functional and/or structural role. The predicted adverse effects on the protein structure, including a reduction of stability and introduction of a helix-to-coil transition, indicated that such a substitution might distort the conformation of the Fks1 protein. We speculate that the conformational change caused by the S656P mutation may reduce drug-target binding affinity, which leads to the pan-echinocandin resistance. Indeed, our experimental results supported the function of S656P towards resistance in *C. parapsilosis*, as well as its functional universality in two other *C. parapsilosis* subspecies.

Moreover, this alteration was found by our study to impact the previously demonstrated response in which inhibition of the β-1,3-D-glucan synthesis with echinocandins leads to a stimulation of the formation of chitin; in this response, the compensatory increase in this polysaccharide partially rescues yeast cells from cell wall damage [39]. Interestingly, these *C. parapsilosis* strains without the S656P alteration produced more Fks1 and chitin to cope with treatment with sub-MICs of micafungin. Strains with the S656P mutation did not demonstrate further compensatory increases in the expression levels of either *FKS1* or *CHS3* upon exposure to micafungin. This result further suggests that the S656P alteration leads to a decreased affinity of Fks1 for echinocandins, and that this decrease is sufficient for Fks1 to antagonize the noncompetitive inhibition of echinocandins at sub-MIC. Further studies are needed to determine the high-resolution structure of *C. parapsilosis* Fks1 for in depth explorations of domain function, and to define the drug-binding domain.

In summary, our study described the distribution of susceptibility to echinocandins of *C. parapsilosis* clinical isolates from China. We discovered significantly higher MICs in blood- as well as ICU-derived isolates, underscoring the necessity of rigorous management of the antibiotic use and diligent monitoring of fungal susceptibility. Furthermore, our work identified for the first time, a pan-echinocandin *C. parapsilosis* clinical isolate harbouring S656P in *FKS1* that was the cause of breakthrough IC. We also demonstrated the role of Fks1^S656P^ in resistance towards all echinocandins and its universality in the *C. parapsilosis* complex.

## Supplementary Material

Supplemental Material
