# Supplementary material for: Decreased echinocandin susceptibility in Candida parapsilosis causing candidemia and emergence of a pan-echinocandin resistant case in China
Source: Emerg Microbes Infect. 2022 Dec 24;12(1):2153086. doi: 10.1080/22221751.2022.2153086 (PMC9793909; doi:10.1080/22221751.2022.2153086)

Figure S1. Phenotypes of the *C. parapsilosis* clinical isolate TJ1197 compared to ATCC 22019. A) Colony morphology. B) Cell morphology. C) Clumping of yeast cells in liquid culture. D) Agar invasion. E) Biofilm formation. ****, *P*<0.0001.


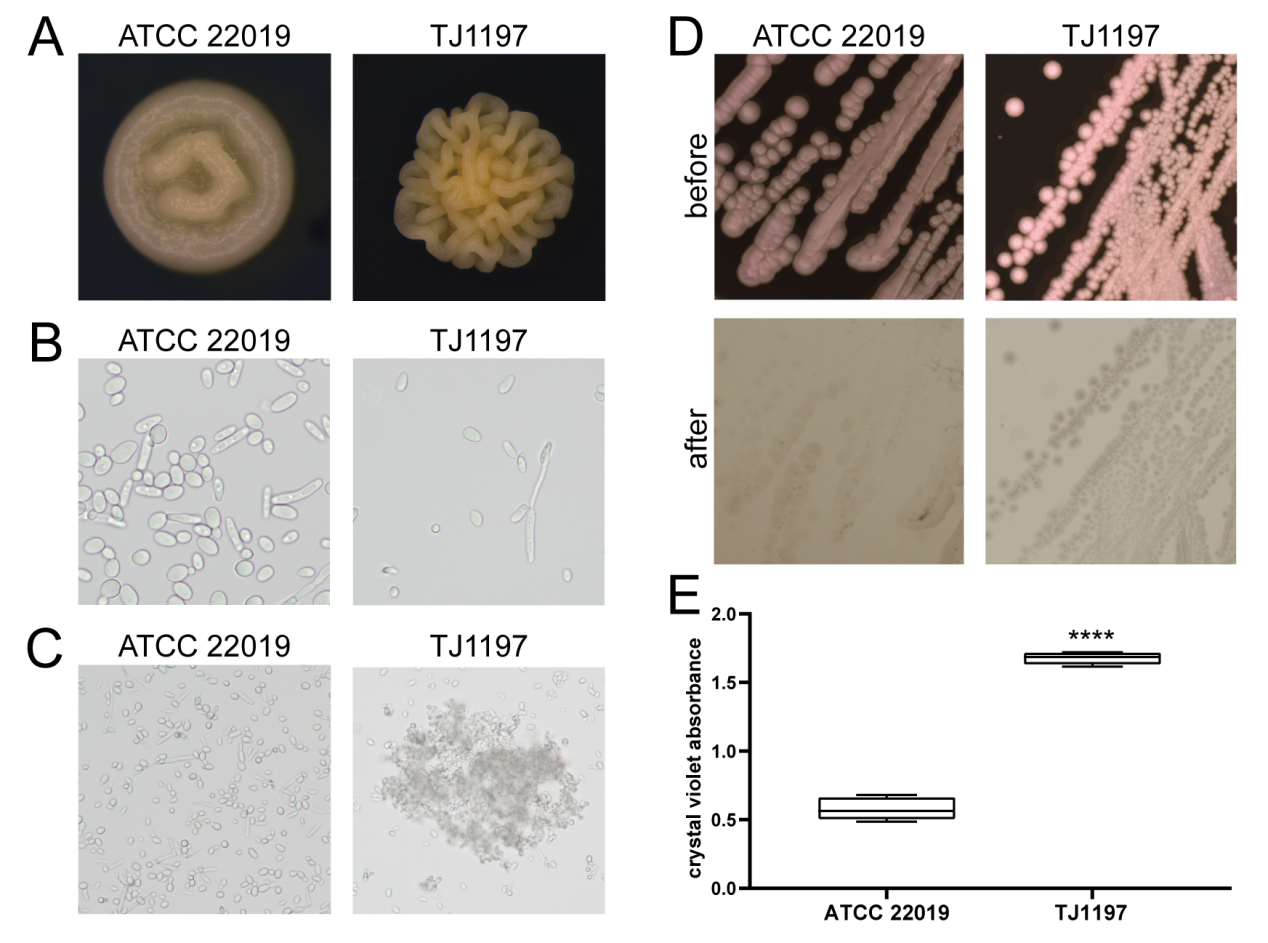

Supplement: Supplemental Material [file TEMI_A_2153086_SM4725.zip › Clean version_Figure S1.docx]
