# Supplementary material for: Decreased echinocandin susceptibility in Candida parapsilosis causing candidemia and emergence of a pan-echinocandin resistant case in China
Source: Emerg Microbes Infect. 2022 Dec 24;12(1):2153086. doi: 10.1080/22221751.2022.2153086 (PMC9793909; doi:10.1080/22221751.2022.2153086)
Supplement: Supplemental Material [file TEMI_A_2153086_SM4725.zip › Clean version_Table S1.docx]

| **Name** | | **Sequence(5'-3')** |
| --- | --- | --- |
| **Generation of the guide RNA** | | |
|  | Cpa_sgRNA_mut/wt_F | CCAAAGATTCAACCAACTTTGCT |
|  | Cpa_sgRNA_mut/wt_R | AACAGCAAAGTTGGTTGAATCTT |
|  | Cor_sgRNA_F | CCACTTGGTTTTTCTTGCCAAGT |
|  | Cor_sgRNA_R | AACACTTGGCAAGAAAAACCAAG |
|  | Cme_sgRNA_F | CCATGTCATTGAGAGATGCCATT |
|  | Cme_sgRNA_R | AACAATGGCATCTCTCAATGACA |
| **Repair templates** | | |
|  | Cpa_RT_mut/wt_F | GGATTGGACATGTGGATGTCGTATTTGTTGTGGGTCTTGGTTTTTCTAGCAAAGTTGGTT |
|  | Cpa_RT_wt_R | TAGCATCTCTCAATGGCAAAGTCAAGAAGAAATAAGATTCAACCAACTTTGCTAGAAAAA |
|  | Cpa_RT_mut_R | TAGCATCTCTCAATGACAAAGTCAAGAAGAAATAAGATTCAACCAACTTTGCTAGAAAAA |
|  | Cor_RT_mut_F | GTCTTACTTGTTGTGGGTCTTGGTTTTTCTTGCCAAGTTAGTTGAATCTTATTTCTTCTT |
|  | Cor_RT_mut_R | CTTTGACAAATTTCTGATTGCATCTCTCAATGGCAAAGTCAAGAAGAAATAAGATTCAAC |
|  | Cme_RT_mut_F | CTAAATTGGTTGAATCTTATTTCTTCTTAACCTTGCCATTGAGAGATGCCATTAGAAACT |
|  | Cme_RT_mut_R | TACCAAACCTCACCAGTACATCTCATTGTGGTCTTTGACAAGTTTCTAATGGCATCTCTC |
| **Colony PCR and Sequencing** | | |
|  | Cpa_Seq_F | GTATTATTGCCGCATTCCTCCA |
|  | Cpa_Seq_R | GTTCTCTGTACATGGAGATAACAA |
|  | Cor_Seq_F | TATCATCACACACTTTCACGGC |
|  | Cor_Seq_R | GGTTGGTGCTCTCAAAGTCC |
|  | Cme_Seq_F | GATCTTTGTGGTGATTTTGGCAC |
|  | Cme_Seq_R | ATGGCTAACAAATGTTCTCTGTAC |
| **qPCR** | | |
|  | Cpa_FKS1_qF | CACCAGCAACCTTATGATATGG |
|  | Cpa_FKS1_qR | AGATGGAGTGTATTGGGTTCCA |
|  | Cpa_CHS3-qF | GTGCATTTGTGGCGTATTTGAC |
|  | Cpa_CHS3-qR | CAACACATTTGAACCAGCACCA |
|  | Cpa_ACT1_qF | ATGCAAACCTCATCACAATCATCT |
|  | Cpa_ACT1_qR | GATAGAGTTGAAAGTAGTTTGGTC |

Table S1. Oligonucleotide sequences used in this study
